# Identification and Functional Analysis of a Protein Disulfide Isomerase (*At*PDI1) in *Arabidopsis thaliana*

**DOI:** 10.3389/fpls.2018.00913

**Published:** 2018-07-19

**Authors:** Zhengrong Zhang, Xin Liu, Rong Li, Li Yuan, Yaqing Dai, Xiaoyun Wang

**Affiliations:** State Key Laboratory of Crop Biology, College of Life Sciences, Shandong Agricultural University, Shandong, China

**Keywords:** PDI, isomerase activities, stress resistance, critical amino acids, endoplasmic reticulum

## Abstract

Protein disulfide isomerase (PDI) catalyzes the conversion of thiol-disulfide and plays an important role in various physiological events in animals. A PDI (*Oa*PDI) from a tropical plant was detailed studied and it was found to be involved in response of biotic stress (Gruber et al., 2007). However, the activities of PDI related to physiological functions in plants are poorly understood. In the present study, a homolog of human PDI in *Arabidopsis* (*At*PDI1), encoded by the gene (*At3g54960*), was characterized. The recombinant *At*PDI1 protein had disulfide isomerase activity *in vitro* and two pairs of conservative cysteines in catalytic domains play a crucial role in the PDI activities. Expression of *At*PDI1 in *Escherichia coli* significantly enhanced stress tolerance of cells and the mutations of critical cysteines almost lose this function. In plants, *AtPDI1* was strongly induced by abiotic stresses and exogenous abscisic acid. An *Arabidopsis* *AtPDI1* knockdown mutant (*pdi1*) and overexpression lines of transgenic plants obtained by this investigation were used to further examine the function of *At*PDI1. The mutant line was more sensitive to stresses than the wild-type, while overexpressing *At*PDI1 increased tolerance of seedlings to abiotic stresses, with a higher germination ratio and longer length of roots than the wild-type. Our results suggested *At*PDI1 played roles in anti-stresses in *Arabidopsis*, which relate to the activities of PDI.

## Introduction

Protein disulfide isomerase (PDI, EC 5.3.4.1) is a thiol-disulfide oxidoreductase, catalyzing oxidation, reduction, and isomerization of target proteins in protein folding and unfolding (Wilkinson and Gilbert, 2005; Ali and Mutus, 2014; Meng et al., 2015). Some PDIs also display chaperone activities, helping protein correct folding (Narindrasorasak et al., 2003; Wilkinson and Gilbert, 2005). However, some play opposite functions, for example, human PDI (*h*PDI) catalyzing extensive intermolecular disulfide cross-linking of lysozyme into large, inactive aggregates (Wilkinson and Gilbert, 2005; Khan et al., 2016). As a classical PDI, *h*PDI (Gene ID: 5034) is composed of four modular domains (*a*, *b*, *b*′, *a*′) and endoplasmic reticulum (ER) retention signal (KDEL sequence) at carboxyl terminus (Alanen et al., 2003). The domain *a* and *a*′ are catalytic domains sharing homology to thioredoxin domain with a di-cysteine motif (CXXC motif), respectively, while domain *b* and *b*′ play roles in keeping the tertiary structures of the whole molecule in U-shape (Kemmink et al., 1997; Byrne et al., 2009). Generally, more than a dozen members of PDIs exist in animal cells and some of them are involved in unfolded protein response (UPR) of persistent stress in ER, which is closely related to the development and progression of diseases like diabetes and neurodegenerative diseases (Schröder and Kaufman, 2005; Honjo et al., 2014). Elevated expressions of some PDIs have been found in patients suffering from lung cancer or melanoma (Xu et al., 2012).

Family members of plant PDIs are also identified in the genome in different plants, for example, 12 members for *Oryza sativa*, 9 for *Triticum aestivum* L., 32 in *Brassica rapa* ssp. *pekinensis*, and 22 for *Zea mays* (Houston et al., 2005; D’Aloisio et al., 2010; Onda and Kobori, 2016; Kayum et al., 2017). Similar to how animal PDIs catalyze the exchange of disulfide, plant PDIs are found to be involved in the unfolded and refolded protein response (Takemoto et al., 2002; Lu and Christopher, 2008; Kimura et al., 2015; Onda and Kobori, 2016; Peng et al., 2017), maturation of the embryo sac (Wang et al., 2008), the programmed cell death of endothelial cells in developing seeds, and response to biotic stress (Gruber et al., 2007; Ondzighi and Staehelin, 2008). The multiplicity and structural difference of plants PDIs suggest that they may serve both specialized and overlapping functions.

Though plants do not suffer animal-specific diseases, plants are more susceptible to stresses in the process of growth and development, due to their immobility. Individual plant PDIs are found to be up-regulated under abiotic stresses. PDIs from *Brachypodium distachyon* L. and *Brassica rapa* ssp. *pekinensis* were up-regulated under abiotic stresses, suggesting their involvement in multiple stress responses (Zhu et al., 2014; Kayum et al., 2017). A PDI from *Jatropha curcas* could notably enhance cold resistance of yeast cells (Wang et al., 2015). PDI from *Oldenlandia affinis* (*Oa*PDI), with activity of PDI, is involved in the oxidative folding of a cystine knot defense protein (kalata B1), suggesting *Oa*PDI takes part in antibiotic stress (Gruber et al., 2007).

As a model plant, *Arabidopsis thaliana* has 12 or more PDI-related members (Houston et al., 2005; Lu and Christopher, 2008). *Arabidopsis* PDIs are classified into three groups based on polypeptide length, presence of signal peptide and ER retention signal, and composition of thioredoxin domains (Lu and Christopher, 2008). There are three members group III (*AtPDI9*–*AtPDI11*) and two members in group I (*AtPDI7*, *AtPDI12*), all of them lacking *b* and *b*′ domains. Group II is the largest group with six members, all of them containing classical four domains. *At*PDI8 contains only three domains (*a*, *b*, *b*′), not belonging to any of the above groups.

As a member in group II, *At*PDI1, which contains classical four domains (*a*, *b*, *b*′, *a*′) with C-terminal ER-anchoring signal. Different from *h*PDI, *At*PDI1 has an additional sequence at N-terminus which is predicted as possible signal peptide targeting to chloroplast. GFP-fused experimental evidences showed that *At*PDI1 is located in ER (Yuen et al., 2013), while *At*PDI1 is also identified in chloroplast by 2D-proteome (Armbruster et al., 2009; Kieselbach, 2013). Up-regulation of *At*PDI1 induced by stress suggested it may play roles in stress responses (Lu and Christopher, 2008). Whether the potential anti-stress function of *At*PDI1 is related to its activity of PDI is unknown. In this study, the purified recombinant *At*PDI1 and cysteine-mutations were used to examine enzymatic activity. To further investigate the function in plants, overexpression lines of *At*PDI1 were obtained by transgenic technique. Different responses to stresses were observed among wild-type, overexpression lines, and a knockdown mutant (*pdi1*). This study will provide a clue about the function of the *At*PDI1 in anti-stresses.

## Materials and Methods

### Bioinformatics Analyses of *At*PDI1

The sequences of *At*PDI1, plant PDIs, and *h*PDI were obtained from the NCBI database^1^. Multiple sequence alignment was performed with CLC sequence viewer. The three-dimensional structure of *At*PDI1 was obtained by homology modeling using the website SWISS-MODEL^2^. The crystal structure of the *h*PDI was downloaded from the PDB database^3^. All structure images were output by PyMOL software.

### Cloning of *At*PDI1 cDNA and Construction of Expression Plasmids

The cDNA sequence of *At*PDI1 was amplified by the RT-PCR method, using *Arabidopsis* leaves treated by NaCl. The sequence was inserted into pET-30a expression plasmid which contained His-tagged. Six mutations of conserved cysteines were amplified using the recombinant vector as the template, which change cysteine to alanine at positions 128, 131, 467, and 470 of *At*PDI1 amino acid sequence. All the primers used in this investigation were listed in Supplementary Table S1. All recombinant plasmid were confirmed by Shanghai Sunny Biotechnology Co. (Shanghai, China), and the recombinant vectors were transformed into *Escherichia coli* BL21 cell.

### Expression and Purification of Recombinant Proteins

Transformed *E. coli* of BL21 cell were grown until OD_600_ of 0.4–0.6, induced by 0.5 mM isopropyl-β-D-thiogalactopyranoside (IPTG) for 2–4 h at 37°C, as done in similar studies (Alergand et al., 2006; Gruber et al., 2007; Wittenberg et al., 2014). The soluble fraction of cells by ultrasonication was purified by affinity chromatography (Ni^2+^-Sepharose) using 150 mM imidazole as elution buffer (Fu et al., 2008). Purity and molecular mass of the *At*PDI1 proteins was confirmed using SDS–PAGE (Supplementary Figure S1). The protein concentrations were determined using a NanoDrop Spectrometer (ND-1000 Spectrophotometer, Peqlab).

### Assay Protein Disulfide Isomerase of Recombinant Proteins

The disulfide bond isomerase activity of recombinant proteins was measured as described previously (Walker et al., 1996). The denatured RNase (reduced RNase) was prepared as described previously (Shin and Scheraga, 2000; Fu and Zhu, 2009). Scrambled RNase A (20 μg/mL) was incubated with or without recombinant proteins into 1 mL solution containing 1 mM GSH, 0.2 mM GSSG, 0.5 mM cCMP, 2 mM EDTA, and 100 mM Tris–HCl performed at 25°C. The formation of active RNase was measured spectrophotometrically by continuous monitoring hydrolysis of the RNase substrate, cCMP, at 296 nm (Walker et al., 1996).

### Cell Growth in Stress Medium

Survival test on LB solid medium was carried out to ascertain the function of *At*PDI1 in *E. coli*. Transformed *E. coli* of BL21 cell were grown till OD_600_ of 0.6, induced by 0.5 mM IPTG for 2 h at 37°C (Du et al., 2014). Cultures were diluted to OD_600_ of 0.6, and then diluted to 10^-2^, 10^-3^, and 10^-4^. For stress treatments, different dilution cultures were spotted on LB medium supplemented with 0.5 M NaCl, 0.3 mM H_2_O_2_, and 0.6 M mannitol, and then incubated at 37°C for 16 h.

Growth analysis in LB solution culture was used to further determine cell growth under abiotic stress. Transformed *E. coli* of BL21 cell were grown till OD_600_ of 0.6, induced by 0.5 mM IPTG for 2 h at 37°C and then 100 μl cultures were inoculated in 20 mL LB solution medium containing mannitol, H_2_O_2_ or NaCl, and incubated at 37°C on a rotary shaker (200 rpm). The bacterial suspension was harvested at every 2 h till 12 h and OD_600_ was measured. Each experiment was carried out in three biological replicates.

### Stress Treatments to Plants, RNA Extraction, and Quantitative Real-Time PCR (qRT-PCR) Analysis

Normally grown 3-week-old *Arabidopsis* seedlings were treated with solution containing 10 mM H_2_O_2_, 20% PEG, 200 mM NaCl, or 100 μM exogenous ABA, respectively, for different times, with water at room temperature as a control. The leaves were collected at different treatment times and the total RNA was extracted from the material. Qualified RNA was used to synthesize cDNA with a cDNA synthesis kit. The GAPDH gene from *Arabidopsis* was used as an internal control for expression normalization in the quantitative real-time PCR (qRT-PCR) as similar investigations did and the relative expression levels were calculated by the method of 2^-ΔΔCt^. The specific primers for amplifying GAPDH and *At*PDI1 genes were used for qRT-PCR and are listed in Supplementary Table S1. The relative expression levels of *At*PDI1 in stressed samples (12, 24, and 36 h) were compared to the controls (0 h) with Student’s *t*-test at significance levels of ^∗^*P* < 0.05 and ^∗∗^*P* < 0.01. Each reaction was carried out in three biological replicates.

### Plasmids for Plant Transformation

The full-length sequence of *At*PDI1 was fused to plant transformation plasmid pROKII-GFP. The promoter sequence of *At*PDI1 (2 kb upstream the gene) was cloned and β-glucuronidase (GUS) gene was fused to plant transformation plasmid pBI121. Plant transformation plasmid was transformed into *Agrobacterium* GV3101, which was used for floral dip transformation. Transgenic plants were selected for kanamycin resistance and verified by genomic PCR with specific primers (Supplementary Table S1). The homozygous lines were obtained. For the GUS assays, 2-weeks-old transgenic *Arabidopsis* T3 seedlings were exposed to 100 μM ABA, 20% PEG, 200 mM NaCl or 10 mM H_2_O_2_ for 9 h, as done in similar studies (Jia H. et al., 2015).

### Plant Materials and Growth Conditions

*Arabidopsis thaliana* Columbia ecotype (Col-0) seeds were sown on Murashige and Skoog (MS) agar medium in greenhouse conditions at 22°C with a 16 h light/8 h dark cycle (light intensity of 200 μmol/m^2^/s; relative humidity of 60–75%). Two lines of overexpressing lines and a knockdown *AtPDI1* mutant (*pdi1-1*) by T-DNA insertion line purchased from the *Arabidopsis* biological resource center^4^ were used for analysis of plants under stresses. For growth on 1/2 MS medium, the seeds of wild-type, T-DNA insertion mutant and overexpression lines were surface-sterilized with 70% ethanol and 2.6% bleach for 5 and 10 min, respectively. Then, seeds were washed for more than 10 mins with sterilized water containing detergent Tween-20. The washed seeds were allowed to germinate on 1/2 MS agar medium, or added 0.6 μM ABA, 200 mM NaCl, 300 mM mannitol, and 10 mM H_2_O_2_, respectively. The concentrations of ABA, NaCl, mannitol, and H_2_O_2_ were determined based on the similar investigations (Jia F. et al., 2015; Gallino et al., 2018). The concentrations used in the present study were stronger than general studies (Brini et al., 2007; Wang et al., 2017). In the root length experiment, the washed seeds were laid in 1/2 MS as above described. All the seeds were photographed after 10 days of growth and the seeds germination rate. The seedling root lengths were counted after 10 days of growth. Each experiment was carried out in three biological replicates.

## Results

### Bioinformatics Analyses on Subcellular Location of *At*PDI1

**FIGURE 1:**
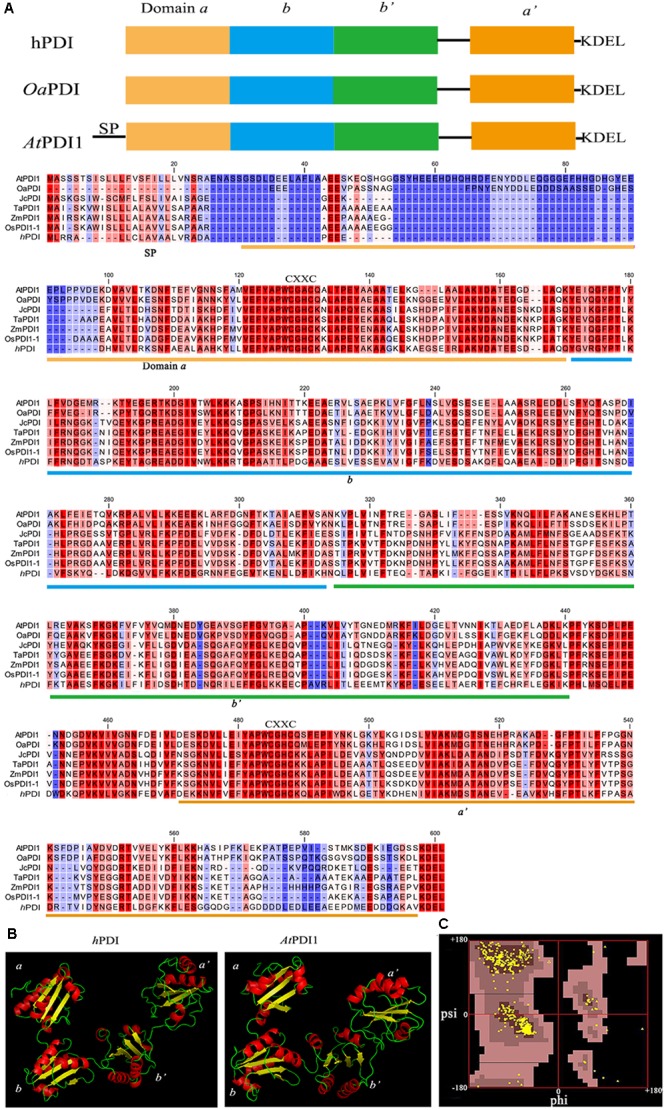
Domain structure and alignment of PDI proteins. **(A)** The domain structures and multiple sequence alignment of *At*PDI1, plant PDIs, and *h*PDI. SP, signal peptide. **(B)** 3D structure of *At*PDI1 constructed by homology-based modeling. **(C)** Ramachandran map of the *At*PDI1 modeling structure, *Phi*, and *Psi* represent the dihedral angle of amino acid residues.

The cDNA sequence of *At*PDI1 (also named as *At*PDIL1–3) was downloaded from the *Arabidopsis* information resource (TAIR) (accession number *At3g54960*), with a complete open reading frame (ORF) of 1740 bp. The ORF encoded a 579-amino acid protein with molecular mass of 64.21 kD and a predicted isoelectric point of 4.74. *At*PDI1 contained the classical four domains (*a*, *b*, *b*′, *a*′) and C-terminal ER-anchoring signal (-KEDL- sequence). Multiple sequence alignment of *At*PDI1 with plant PDIs and *h*PDI showed considerable conserved amino acid sequences, with 50 and 70% similarity of *h*PDI and *Oa*PDI, respectively (**Figure 1A**). However, it was a different case with *Oa*PDI, as *At*PDI1 contains additional sequence in N-terminus, which is predicted as signal peptide. Three-dimensional modeling structure of the *At*PDI1 suggested that four domains (*a*, *b*, *b*′, *a*′) of *At*PDI1 were arranged in the U-shape, similar with other animal and plant classical PDIs. The *a* and *a*′ domains of *At*PDI1 were at the open end of the U-shape with several α helix and β sheets, while *b* and *b*′ were located at the bottom, maintaining the U-shape. The catlytic sites of C-X-X-C motif of *At*PDI1were located in *a* and *a*′ domains, respectively (**Figure 1B**). This modeling structure of *At*PDI1 was reasonable based on checking dihedral angle, since 94% of amino acid residues were located in the energy stable regions, using Ramachandran plot (**Figure 1C**).

As to the subcellular localization of *At*PDI1, we expressed the recombinant plasmids 35S:*At*PDI1-GFP in epidermal cells of tobacco leaf transiently. The GFP signals were almost co-localized with ER-specific label (mCherry) (Supplementary Figure S2), indicating that *At*PDI1 protein was predominantly retained within the ER in epidermal cells of tobacco leaves.

### Recombinant *At*PDI1 Had Activities of Protein Disulfide Isomerase

**FIGURE 2:**
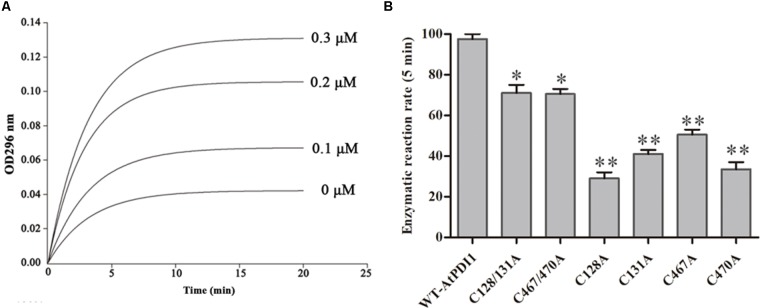
The protein isomerase activity of *At*PDI1. **(A)** Continuous assay of the activities with different concentration of recombinant *At*PDI1. **(B)** Relative isomerase activities of WT–*At*PDI1 and mutations. Results are represented as mean values of enzymatic reaction rate of three biological replicates. Error bars indicate standard deviation and asterisks indicate statistical significance levels (Student’s *t*-test; ^∗^*P* < 0.05; ^∗∗^*P* < 0.01).

The cDNA of *At*PDI1 was successfully cloned by the RT-PCR method, using RNA extracted from leaves treated by NaCl, instead of that from normal growth leaves, which suggested the expression of *AtPDI1* was up-regulated under stresses. The qRT-PCR experiment showed the expression of *AtPDI1* was up-regulated sixfold under NaCl treatment (**Figure 5A**). The purified recombinant *At*PDI1 expressed in *E. coli* was used to examine enzymatic activity by using scramble RNase A. The initial rate of the reaction was promoted as the concentration of *At*PDI1 increased, which was *At*PDI1 concentration dependent (**Figure 2A**). When Cys in thioredoxin motif (Cys-X-X-Cys) are mutated to alanines, the isomerase activities of *At*PDI1 decreased 30–70%, which suggested that four Cys play an important role in the isomerase activities of *At*PDI1 (**Figure 2B**).

### Heterogeneous Expression of *At*PDI1 in *E. coli* Enhanced Cell’s Resistance to Abiotic Stresses

**FIGURE 3:**
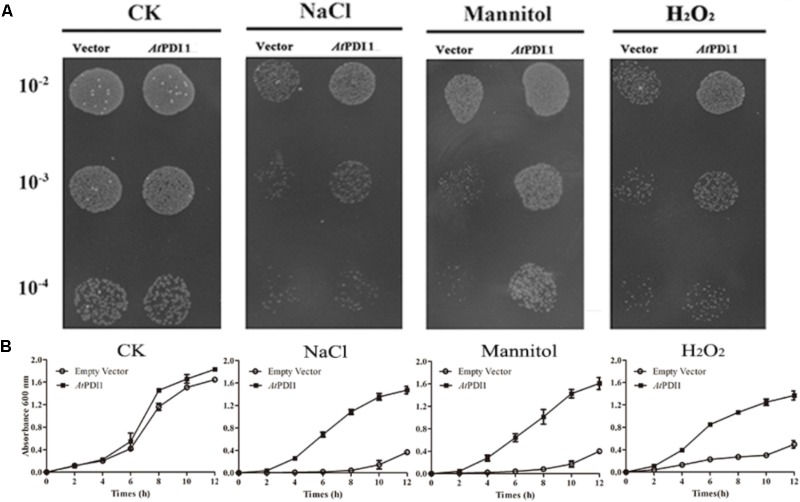
Survival test of *E. coli* cell carrying *At*PDI1 or empty vector on various stress conditions. **(A)** 10 μl cultures from 10^-2^ to 10^-4^ dilutions were spotted on plates treated with various concentrations of NaCl, mannitol or H_2_O_2_. **(B)** *E. coli* cell was cultivated in LB solution medium supplemented with NaCl, mannitol or H_2_O_2_. OD_600_ was recorded at 2 h interval up to 12 h. Results are represented as mean values of three biological replicates and error bars indicate standard deviation.

**FIGURE 4:**
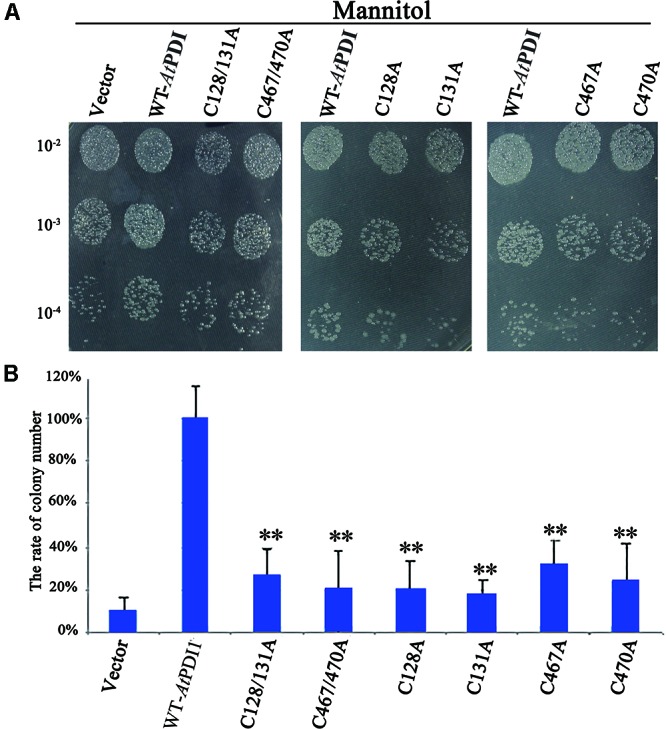
Survival test of *E. coli* cell carrying *AtPDI1*, mutant *AtPDI1* or empty vector on stress conditions. **(A)** 10 μ l cultures from 10^-2^ to 10^-4^ dilutions were spotted on plates treated with mannitol. **(B)** The colony numbers appearing on stress medium were counted in 10^-4^ concentration. Each experiment was carried out in three biological replicates. Results are represented as mean values of three independent replicates and error bars indicate standard deviation. Asterisks show statistical significance levels (Student’s *t*-test; ^∗^*P* < 0.05; ^∗∗^*P* < 0.01).

The stress medium containing different concentrations of osmolytes or H_2_O_2_ was used to check the resistance of *E. coli* cells transformed the plasmid containing *At*PDI1 gene, the empty vector as the controls. No significant differences were observed regarding the growth of the cells on non-stress medium among *At*PDI1 and the empty vector. On the medium with NaCl, mannitol or H_2_O_2_, the colony number showed significant differences, much more colonies with *At*PDI1 than that with empty vector, when same concentration of cultures were inoculated on stress medium plates, respectively (**Figure 3A**). In liquid fermentation, the growth rate of the *E. coli* with *At*PDI1 also remarkably outperformed the control, the time to reach the logarithmic growth phase was much shorter than that with vector (**Figure 3B**). The results suggested that *At*PDI1 conferred strong stress-tolerance to cells against abiotic stresses. Defective mutations of cysteines of *At*PDI1 almost lose the anti-stress activities (**Figure 4**), which suggested that *At*PDI1 isomerase activity and anti-stress function were closely related.

### The Expression of *At*PDI1 Was Up-Regulated by Stresses in Plants

**FIGURE 5:**
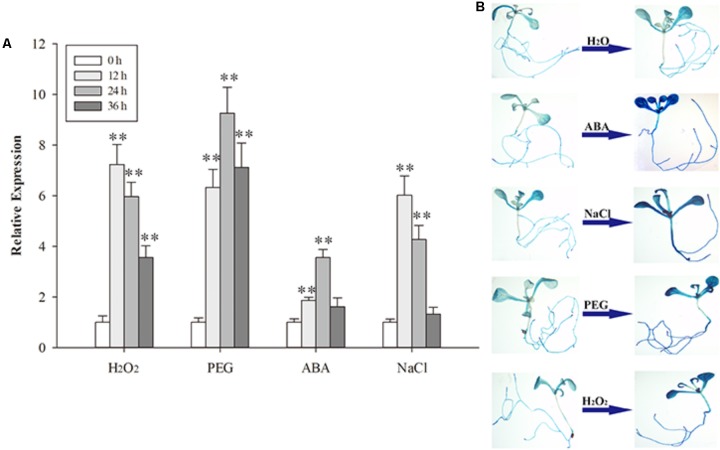
The expression of *At*PDI1 under different stresses **(A)** and the *At*PDI1 promoter-glucuronidase (GUS) expression pattern in transgenic *Arabidopsis* plants under various stresses **(B)**. For **(A)**, results are represented as mean values of three independent replicates and error bars indicate standard deviation. The statistical significance level is shown using asterisks (Student’s *t*-test; ^∗^*P* < 0.05; ^∗∗^*P* < 0.01).

To determine the response of *At*PDI1 in plants, 3-week-old *Arabidopsis* seedlings were treated with H_2_O_2_, PEG, NaCl or exogenous ABA, respectively. The expression of *AtPDI1*, checked by qRT-PCR, was up-regulated under different stresses, approximately four- to ninefold compared with control condition in leaves (**Figure 5A**). The expression peak of *At*PDI1 was reached at 12–24 h under different treatments.

To further test the response of the *At*PDI1 to stresses, the promoter sequence of *AtPDI1* (2 kb upstream the gene) was cloned and linked with GUS gene and transformed into *Arabidopsis*. Four independent transgenic *Arabidopsis* T3 lines were used for GUS histochemical staining assays. The GUS signal of seedlings were significantly enhanced after NaCl, PEG or H_2_O_2_ treatments, respectively, compared with H_2_O treatment (**Figure 5B**). Both the above results suggest that *AtPDI1* was a stress-inducible gene and up-regulated expression may participate in defensive responses to stresses.

### Up- and Down-Expression of *At*PDI1 Affected Abilities of Anti-resistance of Seedlings

**FIGURE 6:**
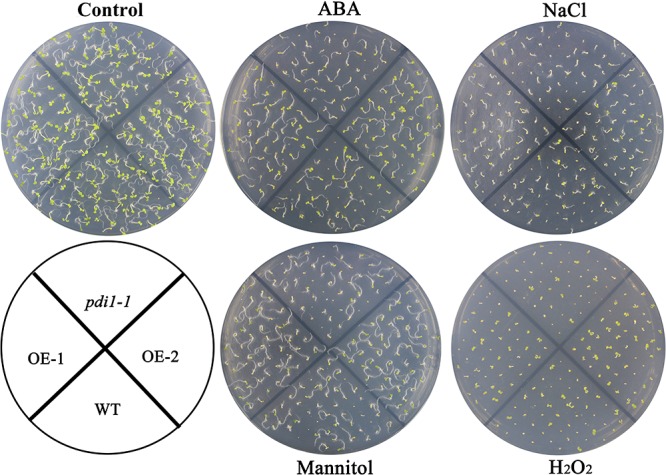
Germination phenotype of the wild type (WT), *pdi*, and *At*PDI1-overexpressing lines under 1/2 MS medium containing ABA (0.6 μM), H_2_O_2_ (10 mM), NaCl (200 mM), or mannitol (300 mM), respectively. Three independent experiments were conducted and each phenotype included 50 seeds.

**FIGURE 7:**
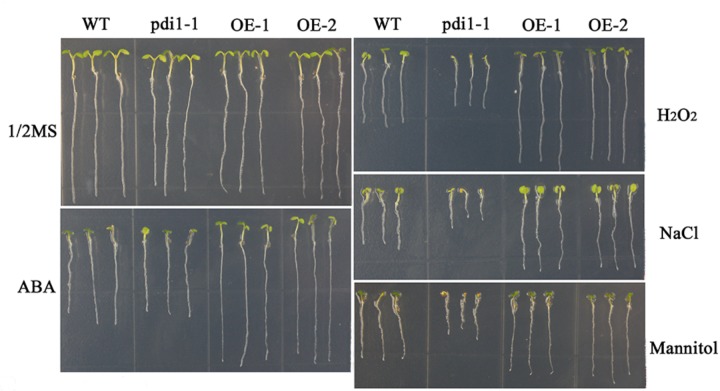
The roots lengths of WT, *pdi*, and *At*PDI1-transgenic lines in stress-free (Control) or stress-containing MS medium. The root lengths of wild-type, *pdi*, and *At*PDI1 transgenic seedlings were determined after 14 days on basal medium supplemented with ABA, H_2_O_2_, NaCl, or mannitol. Each experiment was carried out in three biological replicates.

Independent T3 transgenic plants of overexpressed *At*PDI1 driven by the CaMV 35S promoter were generated. A knockdown *AtPDI1* mutant (*pdi1-1*) by T-DNA insertion line was purchased from the *Arabidopsis* biological resource center^5^. Under normal conditions, no significant differences were observed in the growth among the wild-type, transgenic, and *pdi1-1* plants (**Figure 6**). However, under stress treatments (0.6 μM ABA, 200 mM NaCl, 300 mM mannitol, and 10 mM H_2_O_2_), the seeds of the overexpression lines (OE1 and OE2) germinated much earlier and showed significantly higher germination rates than that of wild type (WT), while the *pdi1-1* mutant was later and lower than that of WT under same stress treatments (**Figure 6** and Supplementary Figure S3). Similar tendency of root length of young seedlings were observed among OE lines, WT, and *pdi1-1* mutant (**Figure 7** and Supplementary Figure S4). However, no obvious differences of phenotype were observed among OE lines, WT, and *pdi1-1* mutant at mature growth stages under stresses (data was not shown). The results showed that *At*PDI1 play important roles in stress-tolerance during seed germination and the roots growth of young seedlings.

## Discussion

When animals and plants are exposed to various stresses, misfolded or unfolded proteins are accumulated, while a series of signal transduction pathways are activated, such as UPR to response the stress (Schröder and Kaufman, 2005). In animals, PDIs existing in ER are key proteins during UPR (Schröder and Kaufman, 2005; Ali and Mutus, 2014). In plants, the relations between ER stress and PDIs is poorly studied. Lu and Christopher (2008) treated seedlings with chemical inducers of ER stress and found that six of twelve *Arabidopsis* PDIs, including *At*PDI1, were up-regulated. Three of them (*At*PDI1, *At*PDI5, and *At*PDI6) have classical constitution of domains (*a*, *b*, *b*′, *a*′) (Supplementary Figure S5). No homologous *b* and *b*′ fold domain could be found in the other three members (*At*PDI9, *At*PDI10, and *At*PDI11). All six members have signal peptide sequence at N-terminus and predominantly retained within the ER (Supplementary Figure S5), while they belong to different groups (Lu and Christopher, 2008). Our results showed that *At*PDI1 predominantly existed in ER and can catalyze disulfide bond formation, which suggested that it played roles in UPR. The T-DNA knockout *Arabidopsis* mutant (*pdi1-1*) was sensitive to stresses, but overexpression *At*PDI1 in WT could significantly enhance the stress resistance of plants. Discovering which proteins interact with *At*PDI1 and then alleviate the protein damages of stress-elicited oxidative stress needs to be elucidated.

Proteomic analyses of chloroplasts showed that *At*PDI1 was also localized in the chloroplast (Wittenberg and Danon, 2008). In chloroplast, there are at least one third proteins whose activities are regulated by thiol-disulfide conversions (Kieselbach, 2013). Compared with intensive studies about reduction of disulfide bonds in chloroplasts, only one thylakoid protein vitamin K epoxide reductase (*At*VKOR/LTO1) has been demonstrated to catalyze the formation of the disulfide bonds in luminal proteins (Feng et al., 2011; Lu et al., 2011; Kieselbach, 2013). However, in chloroplast stroma, which protein catalyzes the formation of the disulfide bond has been unclear until now. *At*PDI1 is regarded as the most likely candidate (Kieselbach, 2013). In this study, *At*PDI1 was confirmed to have activities of PDI and two pairs of cysteins in domain *a* and *a*′ were critical for catalyzing function, just like other classical animal and plant PDIs. *At*PDI1 may have alternative form without ER retention signal by different edit processing according to the National Center for Biotechnology Information (NCBI). However, our results showed that this form of *At*PDI1 was still predominately targeted to ER (Supplementary Figure S2). Whether *At*PDI1 play important roles in chloroplast needs to be elucidated.

In summary, *At*PDI1 was involved in the abiotic stress processes, which was related to its activity of disulfide isomerase. The mechanisms of *At*PDI1 used to regulate anti-stress ability and related physiological functions have yet to be investigated.

## Author Contributions

XW, ZZ, and XL designed the experiments. ZZ, XL, RL, and LY performed the experiments. XL, RL, and YD analyzed the data. XW, XL, and ZZ wrote the paper. All authors read and approved the manuscript.

## Conflict of Interest Statement

The authors declare that the research was conducted in the absence of any commercial or financial relationships that could be construed as a potential conflict of interest.

## Footnotes

1 https://www.ncbi.nlm.nih.gov/gene/

2 https://www.swissmodel.expasy.org/

3 http://www.rcsb.org/pdb/home/home.do

4 https://www.arabidopsis.org/abrc/index.jsp

5 https://www.arabidopsis.org/abrc/index.jsp
