# Supplementary material for: Identification and Functional Analysis of a Protein Disulfide Isomerase (AtPDI1) in Arabidopsis thaliana
Source: Front Plant Sci. 2018 Jul 19;9:913. doi: 10.3389/fpls.2018.00913 (PMC6060501; doi:10.3389/fpls.2018.00913)
Supplement: Supplementary file 1 [file Table_1.docx]

| Primer designations | Primer Sequences（5′-3′） |
| --- | --- |
| *At*PDI1-pET-F | CATCCCATGGCTATGGCTTCGTCATCTACAAGTATCTCT |
| *At*PDIL-pET-R | CCGGAATTCTCACAACTCATCCTTGGAACTATCACC |
| q*At*PDI1-F | CAATGAAGATTATGGAGAAG |
| q*At*PDI1-R | GCAAGTGTCTTAATGTTAT |
| m*At*PDI1_C128/131A_-F | TTACGCTCCGTGGTACGGCGCTTATCAGGCTTTGAC |
| m*At*PDI1_C128/131A_-R | TAAGCGCCGTACCACGGAGCGTAAAACTCCACCATC |
| m*At*PDI1_C467/470A_-F | ATATGCTCCTTGGTATGGCCACTACCAATCATTTGA |
| m*At*PDI1_C467/470A_-R | TAGTGGCCATACCAAGGAGCATATATCTCGAGAAGA |
| m*At*PDI1_C128A_-F | TTTTACGCTCCGTGGTACGGCGCTTGTC |
| m*At*PDI1_C128A_-R | TACCACGGAGCGTAAAACTCCACCATCGC |
| m*At*PDI1_C131A_-F | CCGTGGTGCGGCGCTTATCAGGCTTTGA |
| m*At*PDI1_C131A_-R | TAAGCGCCGCACCACGGAGCGTAAAACT |
| m*At*PDI1_C467A_-F | ATATATGCTCCTTGGTATGGCCACTG |
| m*At*PDI1_C467A_-R | TACCAAGGAGCATATATCTCGAGAAGAAC |
| m*At*PDI1_C470A_-F | CTTGGTGTGGCCACTACCAATCATTTG |
| m*At*PDI1_C470A_-R | TAGTGGCCACACCAAGGAGCATATATCTC |
| *At*PDI1-pROKII-F | GCTCTAGAATGGCTTCGTCATCTACAAG |
| *At*PDI1-pROKII-R | CGGGATCCCAACTCATCCTTGGAAC |
| Pro*_A_*_tPDI1_-F | CCCAAGCTTCATATTATTTAACGGATTTTATGG |
| Pro*_At_*_PDI1_-R | AACGGATCCTGTTGTATGTAAGTGTGTGT |
| GAPDH-F | TGGTTGATCTCGTTGTGCAGGTCTC |
| GAPDH-R | GTCAGCCAAGTCAACAACTCTCTC |

Table S1 Primers used for PCR amplification of genes

Legends of supplementary figure

Fig. S1 Recombinant *At*PDI1 in SDS-polyacrylamide gel electrophoresis. (A) Expression. (B) Purification.

Fig. S2 Subcellular localization of *At*PDI1. The transient expression of the 35S: *At*PDI1-GFP constructs was examined in epidermal cells of tobacco leaf at 48 h after transformation.

Fig. S3. The rate of seed germination of wild type (WT), *pdi* and *At*PDI1-overexpressing lines under various stress treatments. Three independent experiments were conducted and each phenotype contained 50 seeds. Results are represented as mean values of three independent replicates and error bars indicate standard deviation. The statistical significance level is shown using asterisks (Student’s *t*-test; **P*<0.05; ***P*<0.01).

Fig. S4. The roots length of wild type (WT), *pdi* and *At*PDI1-overexpressing lines under various stress treatments after 7 d. Three independent experiments were conducted and each phenotype contained 10 seedlings. Results are represented as mean values and error bars indicate standard deviation. The statistical significance level is shown using asterisks (Student’s *t*-test; **P*<0.05; ***P*<0.01).

Fig. S5. The primary structures of up-regulated *At*PDIs after ER stress. SP: signal peptide.
