# Supplementary figures and images for: Identification and Functional Analysis of a Protein Disulfide Isomerase (AtPDI1) in Arabidopsis thaliana
Source: Front Plant Sci. 2018 Jul 19;9:913. doi: 10.3389/fpls.2018.00913 (PMC6060501; doi:10.3389/fpls.2018.00913)

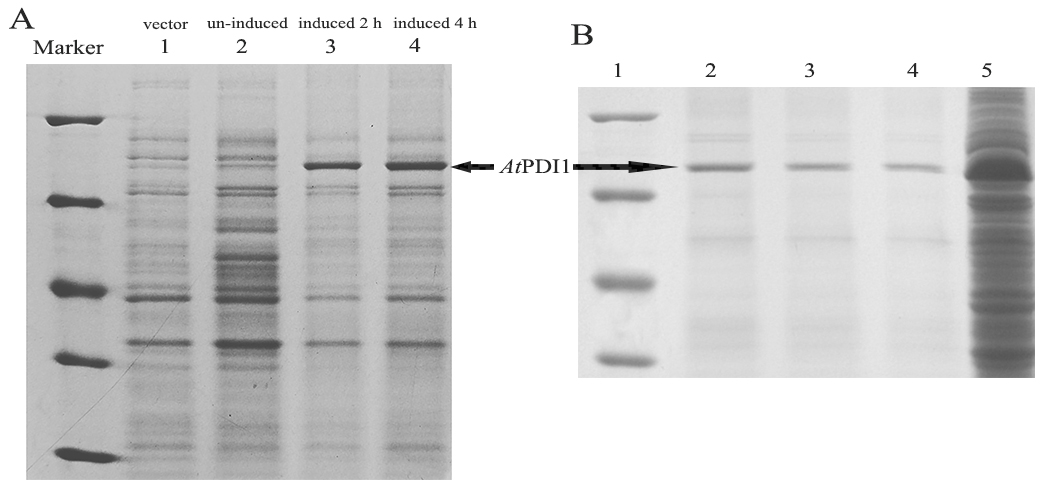

Supplement: Supplementary file 2 [file Image_1.JPEG]

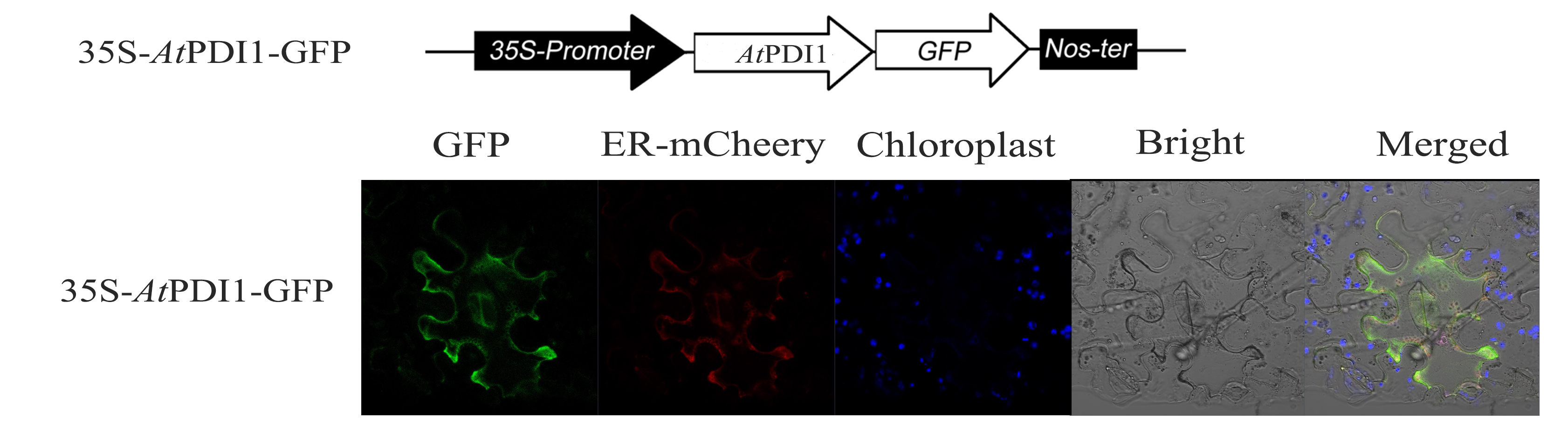

Supplement: Supplementary file 3 [file Image_2.JPEG]

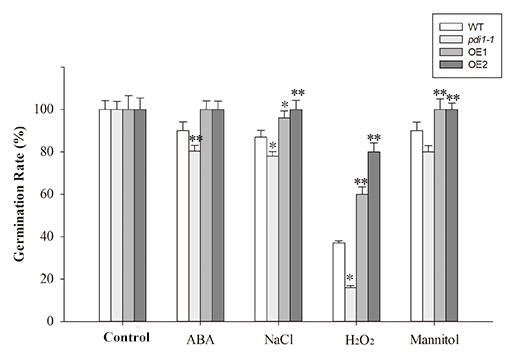

Supplement: Supplementary file 4 [file Image_3.TIF]

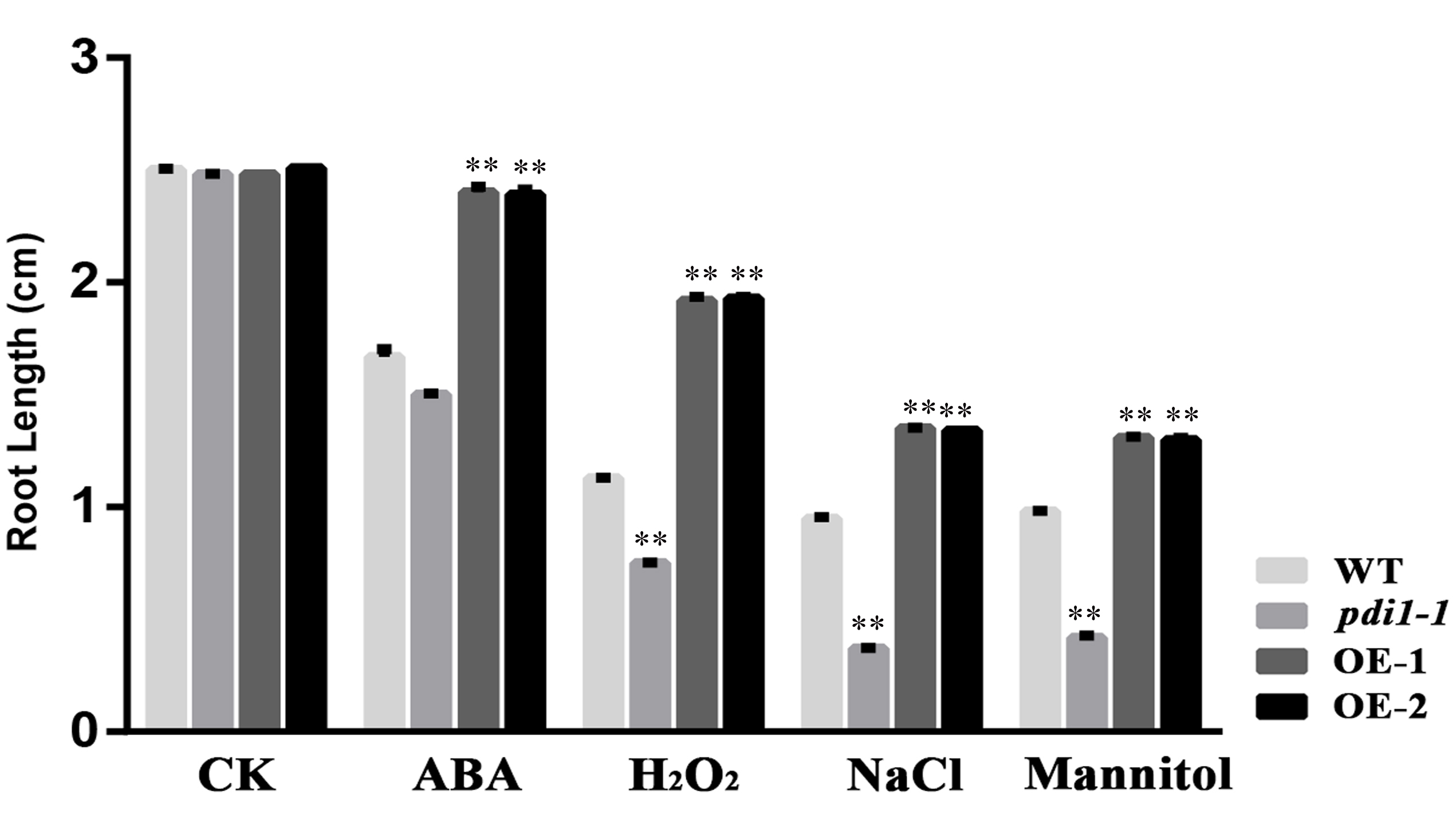

Supplement: Supplementary file 5 [file Image_4.JEPG]

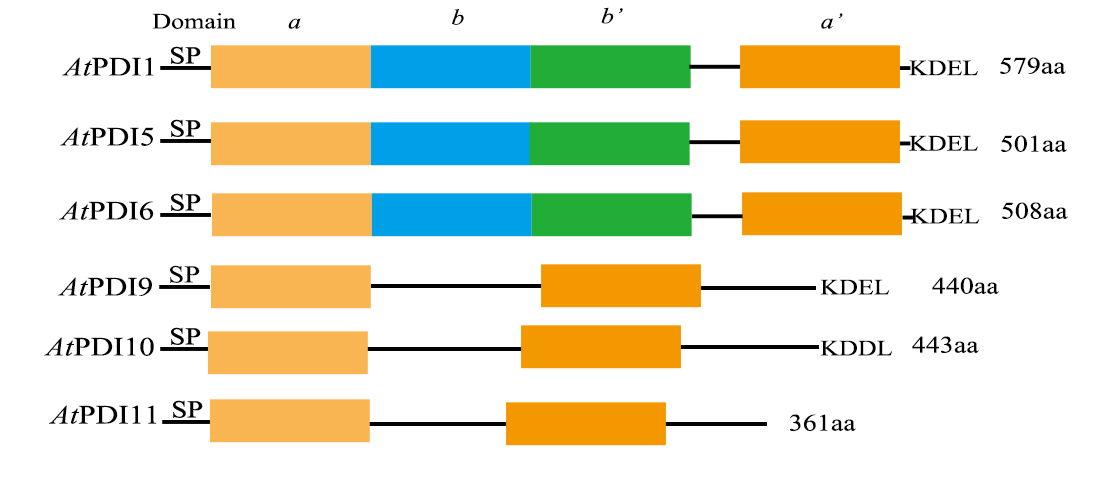

Supplement: Supplementary file 6 [file Image_5.TIF]
